# Association of Pre-Operative Hyponatraemia with Morbidity and Mortality in Patients Undergoing Non-Urgent Degenerative Spine Surgery, a Retrospective Study

**DOI:** 10.3390/healthcare12111140

**Published:** 2024-06-03

**Authors:** Nizar Algarni, Yousef Marwan, Rakan Bokhari, Anas Nooh, Abdullah Addar, Abdullah Alshammari, Musab Alageel, Michael H. Weber

**Affiliations:** 1 Department of Orthopedic Surgery, College of Medicine, King Saud University, Riyadh 12372, Saudi Arabia; aaddar@gmail.com (A.A.); alageelmusab@gmail.com (M.A.); 2 Department of Surgery, College of Medicine, Health Sciences Centre, Kuwait University, Kuwait 24923, Kuwait; yousefmarwan@hotmail.com; 3 Division of Neurosurgery, Department of Surgery, Faculty of Medicine, King Abdulaziz University, Jeddah 21589, Saudi Arabia; rfbokhari@kau.edu.sa; 4 College of Medicine, King Abdulaziz University, Jeddah 21589, Saudi Arabia; anas.h.nooh@gmail.com; 5 Department of Orthopedic Surgery, McGill University Health Centre, Montreal, QC H3J 1A4, Canada; dralshammari.abdullah@gmail.com (A.A.); michael.weber@hotmail.com (M.H.W.)

**Keywords:** hyponatraemia, spine surgery, complications, degenerative, spine surgery, morbidity, mortality

## Abstract

Background and Objectives: Hyponatraemia increases the morbidity and mortality risks of orthopaedic patients. When undergoing spine surgery, hyponatraemic patients have high risks of pneumonia and of staying in hospital for up to 1 day longer compared with non-hyponatraemic patients. This study aims to assess the occurrence of adverse events among patients with pre-operative hyponatraemia after undergoing lumbar surgery. Materials and Methods: A retrospective cohort study was conducted. Patients who underwent spinal surgery in 2011 to 2013 were identified from the American College of Surgeons National Surgical Quality Improvement Program database. Multivariate analysis was conducted to demonstrate the difference in post-operative complication rates between hyponatraemic patients and normonatraemic patients. Post-operative adverse events, need for blood transfusion and length of stay were considered as clinical outcome data. Results: A total of 58,049 patients were included; pre-operatively, 55,012 (94.8%) were normonatraemic and 3037 (5.2%) were hyponatraemic. Multivariate analysis showed that hyponatraemic patients had higher rates of adverse events, blood transfusions and urinary tract infections. Specifically, 632 (20.8%) hyponatraemic patients developed adverse events, compared with 6821 (12.4%) normonatraemic patients; the hyponatraemic patients received transfusions, compared with 6821 (7.4%) normonatraemic patients; and 97 (3.2%) hyponatraemic patients developed urinary tract infections, compared with 715 (1.3%) normonatraemic patients. Finally, an extended length of stay beyond 6 days occurred in 604 (19.9%) hyponatraemic patients, compared with 4676 (8.5%) normonatraemic patients. Conclusions: Our study identified an association between pre-operative hyponatraemia and post-operative adverse events in spinal surgery patients. However, it is unclear whether hyponatraemia caused the higher adverse event rate.

## 1. Introduction

Hyponatraemia is the most common electrolyte abnormality encountered in clinical practice. Up to 42% of hospitalised patients have been found to be affected [1,2,3]. Among this group of patients, a higher cost of care compared with that of other patients has been observed because of prolonged hospital stays, higher peri-operative complication and readmission rates, and increased mortality compared to patients with normal sodium levels [3,4,5,6,7]. 

A large cohort study from the National Surgical Quality Improvement Program (NSQIP) database included all the adults above 18 years old who underwent major surgeries in 2005–2010 [6]; in the mentioned study, they defined hyponatraemia as sodium measurement less than 135 mEq/L. Then, the patients were divided into two groups: hyponatermic and normonatermic. After that, the authors specifically considered the association between pre-operative hyponatraemia and 30-day morbidity and mortality. Pre-operative hyponatraemia was found to predict a higher incidence of wound infections, post-operative pneumonia and major coronary artery events, a finding corroborated by other research [4,5]. This was also true for the orthopaedic surgery subgroup, which showed a higher mortality rate on univariate analysis (odds ratio (OR) = 1.56 [1.22–1.99]) [8,9]. 

Another study found that, among orthopaedic patients admitted to either of two large academic centres, mild hyponatraemia was associated with a 2-fold increased risk of death, whereas severe hyponatraemia exhibited a 4.6-fold increased risk of mortality [10]. In addition, hyponatraemia at the time of presentation was associated with longer hospital stays and a higher likelihood that patients would be discharged to an extended-care facility, such as an acute rehabilitation centre [7].

In the previous studies, the association of pre-operative hyponatraemia with morbidity and mortality has been studied extensively with different surgical categories. However, all of the mentioned studies do not include patients with spinal surgeries. Indeed, few studies have assessed the risk of hyponatraemia for patients who will undergo spine surgery. Those that do exist have concluded that there is a risk of increased hospital stay and risk of pneumonia for these patients [11]. However, other serious adverse events, including death, septic shock, re-intubation, stroke and myocardial infarction, have not yet been assessed.

We aim to address, to the best of the NSQIP’s capability, whether an isolated finding of hyponatraemia in a relatively healthy patient undergoing an elective non-oncological degenerative spinal procedure, with or without fixation, is associated with effects on post-operative events. This sub-population is the major bulk of the surgical caseload in spine surgery practices and the one least vulnerable to developing complications post-operatively. A positive association may have a significant effect on practice since these patients can be optimised in the outpatient setting, potentially with major cost-saving implications. In some institutions, this group of patients might undergo surgery without any pre-operative examination of serum electrolyte levels.

## 2. Materials and Methods

### 2.1. Data Source

We used the American College of Surgeons (ACS)-NSQIP database, which captures data from over 400 academic and private participating centres in the United States and Canada. In the ACS-NSQIP, 300 patient variables were prospectively collected from operative reports, medical records and patient interviews to assess 30-day adjusted surgical outcomes. In this study, patients were identified retrospectively, and they were randomly sampled at eligible hospitals. Clinical data were collected for the entire 30-day post-operative period, regardless of discharge status.

### 2.2. Study Setting and Variables

This study was a retrospective cohort study that reviewed all patients who underwent major spine surgery in 2011–2013; the major spine surgeries include decompression, decompression and fusion of a single vertebra and more. Patients with missing pre-operative data or patients with other electrolyte abnormalities were excluded. We defined hyponatraemia as a sodium level of less than 135 mEq/L. To investigate the association between pre-operative hyponatraemia and post-operative complications, we excluded patients who did not have a recorded pre-operative sodium level from our analysis. The sodium level was defined as the most recent sodium level measured within 90 days of surgery.

Among the variables available in the ACS-NSQIP database, the demographic characteristics of interest included sex, age and race. Co-morbidities included body mass index (BMI; calculated from each patient’s height and weight (kg/m^2^)), a history of diabetes mellitus (recorded as history of type 1 or type 2 diabetes), smoking, dyspnoea (classified as dyspnoea at rest or at moderate excretion), chronic obstructive pulmonary disease congestive heart failure (CHF), dialysis, hypertension, bleeding disorder (haemophilia A, haemophilia B or anaemia), steroid intake for chronic diseases and American Society of Anesthesiologists (ASA) class.

In an attempt to approximate the NSQIP cohort with typical spine practice, we excluded patients diagnosed with malignancies, including blood malignancies (leukaemia, lymphoma or myeloma), those with neurological diseases and those categorised as having emergency surgery. Patients with degenerative changes at the thoracic or cervical levels were also excluded. Patients who underwent lumbar surgery were divided into two groups—namely, the normonatraemic and hyponatraemic groups.

Data on various adverse events in the 30-day post-operative period are explicitly recorded in the ACS-NSQIP based on standard definitions. This study investigates the relationship between a low pre-operative serum sodium level and the occurrence of any adverse events, serious adverse events and minor adverse events. Serious adverse events include death, ventilator support for more than 48 h, re-intubation, stroke/cerebrovascular accident (CVA), deep surgical site infection, cardiac arrest, myocardial infarction, sepsis, pulmonary embolism and return to operating room. Minor adverse events include blood transfusion, urinary tract infection (UTI), pneumonia, superficial surgical site infection and deep venous thromboembolism (DVT). Intraoperative variables of interest include prolonged operative time (>90th percentile) and extended hospital stay (>90th percentile). Post-operative variables of interest include readmission and remaining in the hospital beyond 30 days post-operatively.

### 2.3. Study Ethics

This study was approved by the McGill University Health Center Research Ethics Board to use NSQIP database. The NSQIP database obtained consent for the data sharing for research and quality purposes.

### 2.4. Statistical Analysis

All statistical analyses were conducted using SPSS version 21 (IBM Corp.; Armonk, NY, USA). The Pearson chi-square and student *t*-tests were used to assess the association between patients’ characteristics and sodium levels. Furthermore, multivariate logistic regression was conducted to compare the occurrence of complications between normonatraemic and hyponatraemic patients. All multivariate analyses, controlled for demographic and co-morbidity variables, are included in Table 1. In addition, further sub-analysis was carried out for patients without known co-morbidities who underwent inpatient surgeries and did not have prolonged operative hours.

## 3. Results

### 3.1. Patient Demographics and Clinical Characteristics

A total of 58,049 patients from the NSQIP database who underwent spine surgery between 2011 and 2013 met our inclusion criteria. The overall mean age was 57.6 ± 14.3 years. The sample size was 58,049; of the sample, 49.2% *(n* = 28,560) were men and 50.8% (*n =* 29,489) were women. The average BMI was 30.1 ± 6.6. Fifty-five percent of the population (*n =* 31,926) had an ASA class of 1–2. Of all the patients, 55,012 (94.8%) were normonatraemic, with an average sodium level of 139.5 ± 2.5 SD, and 3037 (5.2%) were hyponatraemic, with an average sodium level of 132 ± 2.1 SD. The demographic, co-morbidity and clinical characteristics of the patients are summarised in Table 1.

A comparison of patients’ characteristics between normonatraemic and hyponatraemic groups revealed significant differences between the cohorts in age, sex and race. Hyponatraemic patients were older, and most of them were female and Caucasian (*p* < 0.001). In terms of patients’ co-morbidities, hyponatraemic patients were more likely to have a history of diabetes, dyspnoea, CHF, renal failure, dialysis, hypertension and steroid intake (*p* < 0.001). In addition, bleeding disorders were more prevalent in the hyponatraemic group (*p* = 0.001), which demonstrated higher rates of pre-operative blood transfusion (*p* < 0.001). Patients in the normonatraemic group were more likely to undergo outpatient surgery (*p* < 0.001). Intraoperatively, higher ASA class (*p* < 0.001) and longer operative time (162.1 ± 107.7 min, *p* < 0.001) were reported for the hyponatraemic group, while the normonatermic group had operative time 149.6 min ± 99.1 (Table 1). The hyponatraemic group had a longer hospital stay by one and a half days on average (*p* < 0.001). Moreover, they had higher rates of return to the operating room and readmission post-operatively (*p* < 0.001; Table 1).

### 3.2. Study Outcomes

The results of the multivariate analyses for adverse outcomes are presented in Table 2. Overall, 7476 (12.9%) patients experienced any type of adverse events, as shown in Table 2. This included 6843 (12.4%) patients in the normonatraemic group and 633 (20.8%) patients in the hyponatraemic group. Multivariate analysis showed that hyponatraemic patients had higher odds of experiencing any adverse event post-operatively (OR = 1.22, *p* < 0.001, 95% confidence interval (CI) 1.10–1.40). Minor adverse events were observed in 5457 (9.9%) patients in the normonatraemic group and 522 (17.2%) patients in the hyponatraemic group (OR = 1.22, *p* = 0.005, 95% CI 1.10–1.40).

The most common minor adverse event was anemia intra- or post-operatively with between one and three packs of red blood cells (Table 3. A total of 4073 (7.4%) patients in the normonatraemic group, as compared to 396 (13.0%) patients in the hyponatraemic group, required a blood transfusion (OR = 1.23, *p* = 0.008, 95% CI 1.10–1.43). In addition, the hyponatraemic group had higher odds of developing post-operative UTI (OR = 1.60, *p* = 0.001, 95% CI 1.20–2.11). Finally, the hyponatraemic group was more likely to remain in the hospital for longer than 6 days post-operatively (OR = 1.52, *p* < 0.001, 95% CI 1.33–1.75).

## 4. Discussion

Our study found out the most common minor adverse event was anemia that required blood transfusion either intra- or post-operatively with between one and three packs of red blood cells. A total of 4073 (7.4%) patients in the normonatraemic group, as compared to 396 (13.0%) patients in the hyponatraemic group, required a blood transfusion (OR = 1.23, *p* = 0.008, 95% CI 1.10–1.43). In addition, the hyponatraemic group had higher odds of developing post-operative UTI (OR = 1.60, *p* = 0.001, 95% CI 1.20–2.11). Additionally, we found out that the hyponatraemic group had a higher risk of a longer hospital stay of more than 6 days post-operatively (OR = 1.52, *p* < 0.001, 95% CI 1.33–1.75).

Peri-operative hyponatraemia is a relatively common health problem in hospitalised patients. Prior studies have shown a high incidence of peri-operative hyponatraemia, in the range of 7.6–26.5% [12,13]. The prevalence of pre-operative hyponatraemia in the current study, which focused on non-urgent spine surgery patients without a diagnosis of cancer, was 5.2%. Patients with pre-operative hyponatraemia can easily go unrecognised and untreated if they are asymptomatic, especially after recent guidelines that recommend against routine laboratory tests in low-risk patients [14]. 

There are multiple factors that contribute to peri-operative hyponatraemia in surgical patients; for instance, ageing is a well-known and well-recognised risk factor for hypo- or hypernatraemia [13]. In addition, excess water (dilution) can be present, either because of administration of a large volume of hypotonic fluids or triggering of the release of antidiuretic hormone by trauma or surgical stress, resulting in water retention [15,16,17,18,19,20,21,22]. There is a well-established association between hyponatraemia and medical co-morbidity, especially in terms of CHF, liver cirrhosis, renal impairment and pneumonia [23]. In addition, syndrome of inappropriate antidiuretic hormone secretion, use of thiazide diuretics and use of selective serotonin reuptake inhibitor antidepressants all have strong associations with hyponatraemia [24,25,26,27,28,29,30]. In this study, some patients were diagnosed with CHF or hypertension and some patients took thiazide and had renal failure.

In our study, pre-operative hyponatraemia was more prevalent among patients with a history of diabetes, dyspnoea, CHF, renal failure, dialysis, hypertension or steroid intake. These are all known risk factors for operative complications and could have confounded our results; therefore, they were controlled for in our analysis. We also excluded cancer patients, since they may have paraneoplastic syndromes; moreover, they may receive intravenous hydration for decreased oral intake, chemotherapy or primary tumour–related major surgery. We excluded urgent cases, since there is not enough time for proper optimisation in these cases, and the patients may differ from the typical elective population in ways we could not correct for in our analysis.

Few studies have examined the relationship between peri-operative hyponatraemia and adverse events in orthopaedic surgery patients [28,29,30]. One study assessed the association of pre-operative hyponatraemia and adverse events in spine surgery patients and found that patients with pre-operative hyponatraemia have a higher risk of prolonged hospitalisation and risk of pneumonia [11]. Our study found similar results in that the patients with hyponatraemia had a statistically significantly higher risk of hospitalisation (*p* < 0.001).

In a large prospective study, Waiker et al. reported an association between hyponatraemia and mortality among orthopaedic surgery patients [3]. In 964,263 patients from the ACS-NSQIP, Leung et al. showed that hyponatraemic patients have greater odds of 30-day mortality; the likelihood of 30-day mortality is higher by 44% and 56% in the whole cohort and in the subgroup of orthopaedic patients, respectively [6]. In this study, our multivariate analysis showed results that partially supported these findings, as patients with pre-operative hyponatraemia had a higher likelihood of experiencing any adverse event in general. However, no significant differences in the odds of severe adverse events between the two groups were found after controlling for co-morbidities and after excluding cancer patients and emergency surgeries. Thus, hyponatraemia may be a marker of co-morbidities and a marker of co-morbidity-associated post-operative complications rather than an independent risk factor for post-operative complications themselves.

We think that we have added to the literature by examining the association of pre-operative hyponatraemia with adverse post-operative events, with the key difference of focusing specifically on pre-operative hyponatraemia and the elective degenerative spine surgery population. As a result, we show a different profile of complications from those reported in other studies that have incorporated all comers. In this paper, we demonstrated that pre-operative hyponatraemia is independently associated with an extended length of stay of 6 days or more. Our finding corroborates the study by Finnian et al., who reported an independent association between hyponatraemia and extended length of hospital stay [14]. In addition, post-operative UTIs and the need for blood transfusion were associated with hyponatraemia among our sample of patients.

The proposed hypotheses to explain a possible causal relationship between hyponatraemia and the considered adverse outcomes are the alteration of the immune function in the presence of hypo-osmolality and increased tissue oedema that may augment stress on wounds, leading to surgical site infection [30,31]. When looking at the surgical site infection, our study found that the incidence of surgical site infection was increased among patients with hyponatraemia, at a rate of 1.4% compared with 0.8% of the normonatraemic patients having superficial surgical site infection. However, the result was not statistically significant (*p* = 0.21).

Patients with fluid and electrolyte issues may be more likely to require prolonged durations of indwelling urinary catheters, which are associated with UTIs. They are also more likely to remain admitted pending correction of their electrolyte imbalances, which could be addressed more cost-effectively in the outpatient setting. However, it has yet to be demonstrated whether hyponatraemia is a causative factor for these complications or just a marker of co-morbidities that are associated with these events.

Previous studies have linked hyponatraemia with several musculoskeletal abnormalities that increase the risk of fracture [24,32,33,34]. Moreover, hyponatraemia has been associated with increased odds of having femoral neck osteoporosis [29]. In addition, Kinsella et al. showed greater odds of experiencing a fracture in hyponatraemic patients, independent of osteoporosis [33]. We were unable to investigate this interesting additional manner by which hyponatraemia may affect long-term outcomes of spine surgery, whether radiologist- or patient-reported, but it would form an interesting hypothesis for future studies to examine in the spine surgery population.

This study is limited by the fact that it is a retrospective study, with data collected from the ACS-NSQIP dataset—that is, a dataset that lacks some variables. Therefore, despite careful statistical analysis to account for differences between the studied cohorts, the lacking variables mean that our results are subject to potential confounders. Moreover, using the ACS-NSQIP dataset restricted post-operative outcome analysis to 30 days post-operatively, because these are the data that were checked and validated in this dataset. Finally, because of the data limitation and the large sample size, the underlying causes of hyponatraemia were not clear.

Despite the limitations outlined above, our study has advantages in that it was the first to specifically look at the elective spine surgery population and explore all adverse events, including severe and minor adverse events. It also controlled for major confounders and co-morbidities in an attempt to tease out the association of isolated hyponatraemia with post-operative events. Despite our rigorous controlling of factors, some minor complications have remained more likely to occur in this population, in which the risk is relatively low. We have identified that pre-operative hyponatraemia in the elective degenerative spine surgery population is indeed associated with increased complications, but these are only minor complications, not major ones. It is also associated with a prolonged length of stay in hospital. These findings may aid the decision-making and risk assessment processes pre-operatively. We recommend further prospective studies to evaluate the long-term adverse effect among patient with pre-operative hyponatraemia.

## 5. Conclusions

In conclusion, our study identified an association between pre-operative hyponatraemia and post-operative adverse events in spinal surgery patients. However, it is unclear whether hyponatraemia caused the higher adverse event rate.

## Figures and Tables

**Table 1:** Study patients’ demographics and their clinical characteristics.

	All Patients (*N =* 58,049)	Normonatremic (*N =* 55,012) Mean (139.5) (±2.5) SD	Hyponatremic (*N =* 3037) Mean (132) (±2.1) SD	*p*-Value
Demographic Characteristics				
Age (year)	57.60 ± 14.31	57.30 ± 14.21	63.71 ± 13.32	<0.001 *
Gender (%)				<0.001 *
Woman	49.2	49.0	53.1	
Men	50.8	51.0	46.9	
Race (%)				0.001 *
Caucasian	82.8	82.7	84.4	
Black or African American	7.3	7.5	5.3	
American Indian or Native	0.4	0.4	0.4	
Native Hawaiian or Pacific Islander	0.2	0.2	0.2	
Asian	1.8	1.8	1.8	
Unknown	7.4	7.4	7.4	
Comorbidities				
BMI (kg/m^2^)	30.13 ± 6.61	30.21 ± 6.61	29.10 ± 6.80	<0.001 *
Diabetes (%)				<0.001 *
Type I	5.4	5.2	9.5	
Type II	11.1	11.0	13.9	
Smoking (%)	23.8	23.7	24.8	0.16
Dyspnea (%)				<0.001 *
At rest	0.4	0.3	1.0	
Moderate exertion	5.6	5.5	8.0	
Pre-operative TIA	0.4	0.4	0.4	0.75
Pre-operative CVA	0.2	0.2	0.3	0.24
COPD (%)	4.3	4.1	8.2	<0.001 *
CHF (%)	0.3	0.2	0.7	<0.001 *
Renal failure (%)	0.1	0.1	0.3	<0.001 *
Dialysis (%)	0.3	0.3	0.7	<0.001 *
Hypertension (%)	51.4	50.3	71.8	<0.001 *
Bleeding disease (%)	1.6	1.6	2.4	0.001 *
Blood transfusion within 48 h preop. (%)	0.3	0.3	0.8	<0.001 *
Steroids (%)	4.0	3.9	5.9	<0.001 *
ASA class (%)				<0.001 *
1	4.7	4.8	1.5	
2	51.0	51.8	36.3	
3	41.7	40.9	55.8	
4	2.6	2.4	6.4	
Clinical Characteristics				
Operation time (min)	150.20 ± 101.2	149.60 ± 99.11	162.10 ± 107.70	<0.001 *
Hospital stays (day)	3.03 ± 5.40	3.00 ± 5.33	4.60 ± 6.44	<0.001 *
Outpatient (%)	21.2	21.5	17.0	<0.001 *
Return to Operation Room (%)	2.7	2.6	4.2	<0.001 *
Readmission (%)	4.9	4.7	7.4	<0.001 *

BMI: Body Mass Index, TIA: A transient ischaemic attack, CVA: Cerebrovascular accident, COPD: Chronic obstructive pulmonary disease, CHF: Congestive Heart Failture, ASA Class: The American Society of Anesthesiologists classification, * Statistically significant (*p* < 0.05).

**Table 2:** Association of Hyponatraemia with Adverse Outcomes in spine surgery (*N =* 58,049).

Outcome	Normonatremic	Hyponatremic	Multivariate Logistic Regression ‡		
			Odds Ratio	95% CI	*p*-Value
Any adverse event	**12.4%**	**20.8%**	**1.22**	**1.10–1.40**	**0.005**
Any severe adverse event	4.2%	7.5%	1.21	0.91–1.60	0.20
Death	0.3%	1.2%	1.53	1.10–2.45	0.08
Ventilator > 48 h	0.4%	0.7%	0.63	0.34–1.20	0.14
Re-intubation	0.4%	1.2%	1.33	0.85–2.11	0.22
Stroke/CVA	0.1%	0.2%	0.41	0.11–1.65	0.20
Deep surgical site Infection	0.5%	0.7%	1.20	0.67–2.10	0.60
Cardiac arrest	0.2%	0.4%	0.74	0.34–1.80	0.50
Myocardial Infarction	0.2%	0.3%	0.61	0.24–1.53	0.30
Sepsis	0.6%	1.1%	1.20	0.72–1.90	0.54
Septic shock	0.2%	0.4%	1.21	0.55–2.44	0.70
Pulmonary Embolism	0.4%	0.6%	1.51	0.91–2.60	0.13
Return to the operation room	2.6%	4.2%	1.03	0.81–1.34	0.84
Any minor adverse event	**9.9%**	**17.2%**	**1.22**	**1.10–1.40**	**0.005**
Anemia	**7.4%**	**13.0%**	**1.23**	**1.10–1.43**	**0.008**
Urinary Tract Infection	**1.3%**	**3.2%**	**1.60**	**1.20–2.11**	**0.001**
Pneumonia	0.7%	1.4%	1.14	0.80–1.71	0.52
Superficial Surgical Site Infection	0.8%	1.4%	1.34	0.91–2.04	0.21
DVT/thrombophlebitis	0.6%	0.7%	0.70	0.41–1.20	0.20
Extended Length of Stay (>6 days) (>90th percentile)	**8.5%**	**19.9%**	**1.52**	**1.33–1.75**	**<0.001**
Operation time (>276 min) (>90th percentile)	9.8%	13.2%	1.12	0.90–2.51	0.10
Remained in the hospital 30 days postop	0.2%	0.4%	0.70	0.31–1.80	0.42
Readmission	4.7%	7.4%	1.21	1.00–1.41	0.14

CVA: Cerebrovascular Accident, DVT: Deep Venous Thrombosis, Values in boldface indicate statistical significance (*p* < 0.05), ‡ Each line represents a separate multivariate logistic regression analysis for each variable and adjusted odds ratio and *p*-value by controlling for all demographics and comorbidities found in Table 1.

**Table 3:** Sub-analysis of patients without known comorbidities who underwent spinal surgery as an inpatient and did not have prolonged operative hours (*N =* 22,662).

Outcome	Normonatremic (*n =* 22,117)	Hyponatremic (*n =* 545)	Multivariate Logistic Regression ‡		
			Odds Ratio	95% CI	*p*-Value
Any adverse event	6.3%	9.9%	1.31	0.93–1.72	0.13
Any severe adverse event	2.5%	3.9%	1.15	0.72–1.84	0.60
Death	0.1%	0.4%	2.84	0.63–12.91	0.21
Ventilator > 48 h	0.1%	0.0%	0.91	0.10–4.41	0.61
Re-intubation	0.2%	0.2%	0.60	0.10–4.41	0.61
Deep surgical site Infection	0.3%	0.2%	0.43	0.10–3.11	0.41
Sepsis	0.3%	0.2%	0.30	0.04–2.21	0.23
Septic shock	0.0%	0.2%	3.12	0.40–26.3	0.31
Pulmonary Embolism	0.2%	0.6%	1.53	0.51–5.03	0.50
Return to the operation room	1.9%	2.8%	1.23	0.71–2.10	0.51
Any minor adverse event	4.3%	7.3%	1.40	0.96–1.94	0.079
**Anaemia**	**2.6%**	**5.3%**	**1.64**	**1.11–2.51**	**0.02**
Urinary Tract Infection	0.7%	1.1%	1.10	0.51–2.42	0.91
Pneumonia	0.2%	0.6%	1.44	0.44–4.70	0.60
Superficial Surgical Site Infection	0.6%	0.6%	0.98	0.31–3.12	0.97
DVT/thrombophlebitis	0.3%	0.4%	0.80	0.20–3.30	0.75
**Extended Length of Stay (>6 days) (>90th percentile)**	**3.9%**	**12.0%**	**2.21**	**1.61–3.00**	**<0.001**
Remained in the hospital 30 days postop	0.1%	0.5%	4.60	1.10–22.10	0.056
Readmission	3.2%	4.9%	1.30	0.91–2.10	0.22

DVT: Deep Venous Thrombosis, Values in boldface indicate statistical significance (*p* < 0.05), ‡ Each line represents a separate multivariate logistic regression analysis for each variable and adjusted odds ratio and *p*-value by controlling for all demographics and comorbidities found in Table 1. Mean OR time for the normonatremic group was 118 ± 59.7 min and for the hyponatremic group was 121.1 ± 60.8 (*p* = 0.24).

## Data Availability

The data used to support the findings of this study are available from the corresponding author upon request.

## Abbreviations

COPD	Chronic obstructive pulmonary disease
CHF	congestive heart failure
TIA	Transient Ischemic Attack
CVA	Cerebral Vascular Accident
BMI	Body Mass Index
DVT	Deep Venous Thrombosis
ASA	American Society of Anaesthesiologists Score
SD	Standard Deviation
